# Natural Pathogen Control Chemistry to Replace Toxic Treatment of Microbes and Biofilm in Cooling Towers

**DOI:** 10.3390/pathogens6020014

**Published:** 2017-03-31

**Authors:** Lon Brouse, Richard Brouse, Daniel Brouse

**Affiliations:** 1 Chemistry Consultant, B.A. Chem., Phys., Math, M.Ed., D.C., 2200 Mead Ln., Montrose, CO 81401, USA; 2 Sunnyside Health Center, 17396 S. Rory Ct., Oregon City, OR 97045, USA; djbrouse@hotmail.com; 3 Southwestern Oregon Community College, B.S., Biochem. & Biophys., D.C., 1448 Evergreen Dr., Mail Stop 2070, Coos Bay, OR 97420, USA; dcbrouse@gmail.com

**Keywords:** pathogens, *Legionella*, amoeba, protozoa, biofilm, antibacterial, antimicrobial, cooling towers, biocides, polyvalent metals

## Abstract

Application of toxic antibacterial agents is considered necessary to control prevalent fresh water microorganisms that grow in evaporative cooling water systems, but can adversely affect the environment and human health. However, natural antibacterial water chemistry has been applied in industrial cooling water systems for over 10 years to inhibit microorganisms with excellent results. The water chemistry method concentrates natural minerals in highly-softened water to produce elevated pH and dissolved solids, while maintaining low calcium and magnesium content. The method provides further benefits in water conservation, and generates a small volume of non-toxic natural salt concentrate for cost efficient separation and disposal if required. This report describes the antimicrobial effects of these chemistry modifications in the cooling water environment and the resultant collective inhibition of microbes, biofilm, and pathogen growth. This article also presents a novel perspective of parasitic microbiome functional relationships, including “Trojan Protozoans” and biofilms, and the function of polyvalent metal ions in the formation and inhibition of biofilms. Reducing global dependence on toxic antibacterial agents discharged to the environment is an emerging concern due to their impact on the natural microbiome, plants, animals and humans. Concurrently, scientists have concluded that discharge of antibacterial agents plays a key role in development of pathogen resistance to antimicrobials as well as antibiotics. Use of natural antibacterial chemistry can play a key role in managing the cooling water environment in a more ecologically sustainable manner.

## 1. Introduction

Removing water or essential organism nutrients will inhibit microbiological growth, as does increasing certain components that negatively shift the chemistry of the organism’s environment [1] (pp. 38–43) [2] (p. 31). In the case of neutrophile organisms, increasing acidic or basic components will inhibit normal metabolism [3,4,5,6]. Thousands of years ago, people of widely diverse cultures discovered that fermentation of fruits and vegetables generates acids, gases, or alcohol; depending on the food product and the specific bacteria, fungus, or mold involved [7] (pp. iv–v). These microbiological metabolic wastes provided a shift in environmental chemistry that accounted for the preservation effects. People also discovered that soap helps to clean surfaces, and due to the high pH from the caustic soda derived from wood ashes (lye soap), also discovered another potent microbiological inhibitor. For over 100 years, caustic solutions have been widely employed to sanitize food processing equipment.

**Figure 1:**
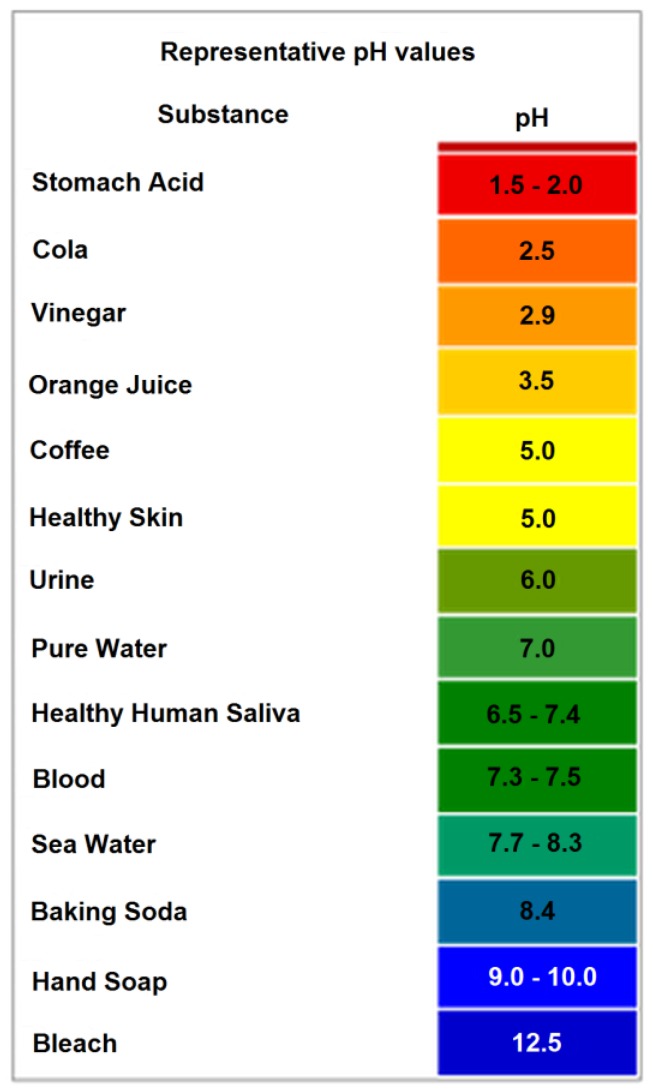
pH values with associated foods, chemicals, and natural environments [5].

The figure below (Figure 1) illustrates the relative acid/neutral/basic pH concentration properties along with some common natural and processed materials, as well as pH of food chemistry. Neutrophile organisms, as indicated by their name, typically only survive in the neutral (6 to 9) pH range. Natural or processed food with a pH outside the neutral range typically inhibits spoilage by microorganisms [5].

Another widespread, historical practice for preserving perishable foods, especially meat and fish of all kinds, was to pack them in salt. This process exerts osmotic pressures on the products and produces naturally dehydrated, well-preserved foods that are inhospitable to microbes that cause spoilage. Salt-packing has continued to be relied upon by modern food processors around the world.

Water-based food products are frequently preserved using salt and either high or low pH solutes that are outside neutrophile microorganism growth ranges [5]. Food processors also use pH-adjusted solutions to sanitize equipment. Such techniques developed over millennia to control microbiological growth have been successfully applied in water-based industrial processes.

Scientists should continue to investigate natural processes as well as historical knowledge in seeking sustainable pathways and solutions for emerging problems, as presented in this report.

Note: The authors use or pair the terms, antibacterial and antimicrobial throughout this review, dependent on the term used by the respective references cited in this review. Antimicrobial is a broader term that can include microbial species not specifically addressed in some discussions.

## 2. Incumbent Cooling Tower Microbe Control Practices and Environmental Impact

The most widely used, anthropogenic, water-based industrial process is evaporative cooling, which impounds warm water along with both air- scrubbed and makeup water nutrients in cooling tower systems. These water conditions provide an excellent environment for micro-organism growth and proliferation. Humans and the environment are in constant contact with these cooling towers, used predominantly for comfort cooling, but also for heat removal in industrial and electric power generation. According to The US Geologic Survey (USGS), in 2010, fresh water use in anthropogenic cooling systems is second only to agricultural irrigation in the US [8]. Traditional methods used to control microbial growth and pathogen potentials in these circulating water systems relied on various antibacterial agents (biocides) to oxidize or metabolically poison these single-celled organisms. However, these antibacterial agents have limited application ranges, deterioration and dissipation rates, and effectiveness.

Two primary classes of antibacterial agents are relied upon in cooling tower systems, and have different limitations. The halogen-based oxidizers are converted into ineffective forms at pH above about 8.5, and dissipate rapidly in the water or are gassed off by tower air scrubbing. Abdel-Nour et al. [9] state that chlorine derivatives are the most common and effective biocides used to control water-based pathogens. According to Jjemba, chlorine-based biocides have been successfully used to control microbiological proliferation in water systems, but only if the free residuals are maintained at 0.5 mg/L or higher. However, excessively high residuals of chlorine derivatives are required to remove biofilm and infection, leading either to corrosion damage of the engineered systems, or exceeding toxic discharge limits. Chloramine is less aggressive to the equipment, but is not effective in eliminating *L. pneumophila* form water-based biofilms [10].

Most non-oxidizers either deteriorate or dissipate rapidly, and are ineffective at high pH. Both of these classes of antimicrobials are regulated to maximum concentration in cooling tower discharge due to toxic environmental impact. This is increasingly impactful when the agents are applied in cooling tower systems operating with high discharge volume at low Cycles-Of-Concentration (COC) due to the water quality properties. Typically driven by hard water scale limitations, cooling towers are operated with high-blowdown water wastage, which dictates use of higher biocide volumes to maintain targeted antibacterial concentrations.

The goal for biocide suppliers has been to find ever more powerful agents that could kill bacteria and other micro-organisms wherever their presence was undesirable. Any man-made antimicrobial chemical capable of the mass destruction of single-celled organisms can and does poison multi-cellular organisms, up to and including humans. The undesirable impact of antimicrobial agents and their byproducts on humans, other species and the environment are forcing us to eliminate as many toxic chemicals from our environment as possible. Once toxic treatment chemicals are added to a water system, they may be difficult to remove, resulting in their passing through typical treatment processes and ultimately discharged back into the natural environment.

## 3. Pathogen Control Effects of Natural Antimicrobial Chemistry in Cooling Towers

With respect to pathogen and *Legionella* risks and control, this review is primarily limited to cooling towers, evaporative condensers and other evaporative cooling water systems. These systems must be addressed separately from other sources of waterborne pathogen exposures due their unique water concentrating chemistry and aerosol drift exposure vectors.

Historical application of antimicrobials to control prevalent fresh water microorganisms was primarily focused on control of biofilm, since biofilm severely limits the heat removal efficiency of cooling equipment. However, the emerging awareness that cooling towers are harbingers and distribution vectors for Legionellosis, has required improved control strategies. According to Jjemba et al. Legionellosis is the most common waterborne disease reported in the US and tracking data shows a steady increase [10]. Abdel-Nour et al. assert that *L. pneumophila* is an aquatic pathogen that is ubiquitously found in nature, in both anthropogenic structures and in environmental waters [9].

Host amoeba and parasitic *Legionella* organisms inside a biofilm may be largely protected from antibacterial agents (including oxidative halogens) due to the extracellular organic material surrounding the organisms. Natural antimicrobial chemistry provides an improved strategy to control biofilm, the host organism, and proliferation of the parasitic organism. The highly-concentrated natural antibacterial chemistry, specifically high in alkalinity (pH) and osmotic (TDS) residuals, results from reduced water wastage from the cooling tower operation. This inhospitable chemistry remains in constant contact with any microorganisms in the cooling water and cannot be avoided as this becomes the organism’s environment. Because they are returned to the environment from which they were derived, concentrated TDS of the commonly occurring natural minerals have no toxicity when subsequently diluted and returned to the environment, in contrast to regulated chemical toxicants. Alternately, when required, the highly concentrated, small volume of high TDS discharge can also be more economically segregated for concentrate disposal.

Microorganisms derived from either fresh water or ”nutrient rich” wastewater sources are equally inhibited with this method, expanding practical alternatives for cooling water supply using various wastewater sources. Jjemba says [10] that recycled water is becoming a necessary addition to fresh water for applications where potable water is not required. However, elevated levels of bacteria, protozoa, along with TDS, organic carbon, nitrogen and phosphorous, provide nutrients for microorganism, and pathogen growth densities that pose challenges for using this valuable resource in anthropogenic water systems. Jjemba also states that the maximum *Legionella* counts were found in cooling water with pH of 8.4 to 9.1. These results are within the neutrophile bacteria range described in this review.

Over the last 10 years, cooling tower operators have used sodium-cycle ion exchange pre-treatment to remove hardness ions, and have then concentrated the cooling tower water to very high COC (high TDS) through evaporation, coupled with almost no tower water wastage or losses. This method produces a natural water chemistry with an alkaline pH, such that the water is antibacterial to the neutrophiles that commonly inhabit cooling tower systems [1]. Likewise, the concentrated natural water chemistry has a high concentration of soluble salts (TDS) which also inhibits microorganisms. The method has been established as highly effective. Exchanging the divalent calcium and magnesium for monovalent sodium, the treated makeup water can be concentrated by tower evaporation up into the 50× to 100× COC range. Removing the divalent ions from the makeup water prevents hard water scale. Only sodium salts remain in the cooling water system, all of which are highly soluble. Low-calcium water has also been studied and shown to inhibit biofilm formation in cooling water systems [2,9,10,11].

Most of the value from water savings is derived in operating cooling tower systems when 10× COC is attained. However, further increasing the natural mineral concentrations by evaporation produces some truly significant benefits. The high-COC buffered carbonate alkalinity produces water in the 9.7 to 10.0 pH range. As previously described, sufficiently elevated pH is an excellent neutrophile inhibitor.

Attrition of antibacterial agent residuals occurs primarily from blowdown in low-COC systems, and thus requires continuous performance monitoring and residual concentration adjustments. During deliberate or unplanned loss of circulation for extended periods, these agents can lose their effectiveness as they dissipate or are unevenly distributed in the system. Such conditions, caused by absence of circulation, are then exacerbated by generating a mechanical aerosol of droplets containing pathogens in tower drift. This combination of operational issues has been reported to be the most common root cause of *Legionella* proliferation and outbreak [2,12] Loss of control of microbiological growth rates also typically results in higher colony-forming unit (CFU) counts or adenosine triphosphate (ATP) concentrations that are reported in relative light units (RLUs).

Concentrating low-level total dissolved minerals (TDS) in the makeup water by 50× to 100× produces a high-TDS water, often in the 10,000 to 100,000 mg/L range. TDS affects the osmotic pressure exerted on the microbiological cells while alkalinity (pH), reacts with cell enzymes and membranes, to prevent cell amplification (propagation) [2,7,13]. According to Rahimian and Anderson, both of these circulating tower water chemistry parameters should be measured and evaluated to maintain their kill synergy [1].

**Figure 2:**
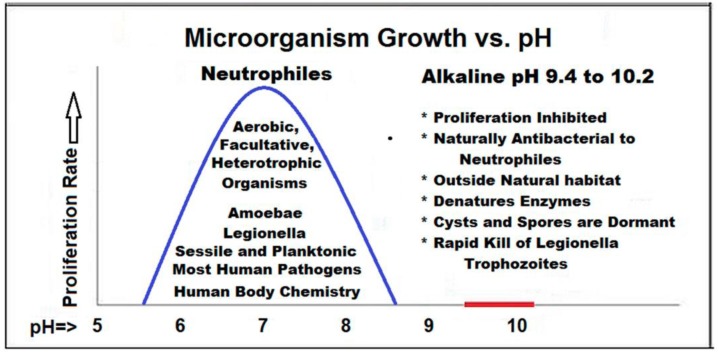
Antimicrobial Effects of shifting pH on neutrophile survival. Neutral pH levels (± 1.5 pH units) are conducive to neutrophile microorganism proliferation. As pH rises, in the 9.0 to 10.0 range, stress increases on neutrophile metabolic processes. (Figure by Lon Brouse).

**Figure 3:**
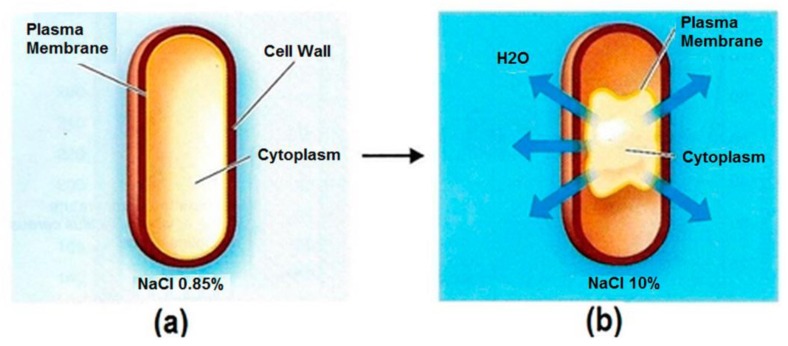
Effects of NaCl concentration on water balance in cells. (**a**) A typical bacterial cell with a semi-permeable membrane in isotonic NaCl solution. The water movement is at equilibrium into and out of the cell. (**b**) A plasmolyzed cell in a higher concentration, hypertonic NaCl solution. The water movement is unbalanced, resulting in cell volume shrinkage and disrupted metabolic activity [14].

The two following diagrams (Figure 2 and Figure 3) depict the effects on neutrophiles imparted by elevated pH and concentrated TDS.

## 4. Validation and Control Testing Methodologies for Cooling Tower Microbes

The Centers for Disease Control and Prevention (CDC), has estimated that there are upwards to 18,000 cases of Legionellosis each year in the U.S. and the Occupational Safety and Health Agency (OSHA) estimates 4000 deaths per year result from Legionnaires’ Disease [15]. It is important for cooling system operators and health care workers to have accurate testing methods to validate *Legionella* infections in water systems so appropriate treatment procedures can be implemented to prevent human disease. These lab tests are not intended to be used in control programs, rather to validate site managed procedures. Accurate validation averts disruption and unnecessary cost for responding to false positive results. McCoy says [16], The CDC laboratory proficiency program (ELITE), laboratories analyze standardized samples of *Legionella* cultures for analysis and return the results to the CDC. Samples with less than 10 CFU/mL of the bacteria are labeled ‘variable’ because this is near the reliable limit of detection for *Legionella*. McCoy [16] states that HACCP programs need to be established with accurate validation methods which provide guidance in implementing effective site pathogen management techniques.

According to Rahimian-Pour and Bertram et al. [1,2], on-site culture media testing, without sample dilution, followed by immediate incubation generates the most consistent culture results [1,2]. Dilution procedures designed to amplify plate count accuracy also eliminate the high pH and TDS inhibiting environment. Some laboratory tests (e.g., BYCE media specifically for *Legionella pneumophila*), are reportedly well-buffered and do not require pre-dilution of the samples, but these tests, like all off-site testing, suffer from potential sample degradation from sample-to-analysis time delays [2]. Additionally, these laboratory tests have not been successfully converted into reliable field tests. Cooling tower monitoring does not focus on *L. pneumophila*, but uses general aerobic indictor organisms for screening. When incubated, culturable microbes as well as accumulated dormant cysts and spores enjoy nearly ideal growing conditions. False high CFU/mL results produced under these conditions can therefore misrepresent the active microbiological population present in the cooling water [1,2].

Under ideal conditions, some bacteria can double their population every 30 min. Any delay in analyzing microbiological results for an operating system can increase the severity of the problem and delay an appropriate response. Growing conditions and the microbiological population are closely linked. Keeping culture testing methods in perspective, according to Kaerberlin, Lewis, and Epstein [17] the majority of culture tests only report 0.1 to 1% of the total population due to the selection pressures generated by the artificial growth environment for the microorganisms to grow [13]. Testing methodologies are improving, but Whiley [18] and Ashbolt report [19] that traditional culture methods were found to positively identify only 34% of known *Legionella pneumophila* samples while a new quantitative polymerase chain reaction (qPCR) test has been shown to accurately detect 72% of the samples. Ashbolt continues to report that the reduced culturability may be due to the bacteria reverting to a cyst-like state, or perhaps the bacteria grow slower on artificial media. The pathogenic cells may be more consistently grown with a co-culture of amoebae [19]. In addition, Joint, Mahling, and Querellou highlight the specific challenges in culturing marine microorganisms, stating an even lower culturable success of only 0.001% to 1% of the assemblage [20]. This is even more important when we are dealing with highly concentrated cooling tower water that approaches, and in some cases exceeds, the TDS of sea water.

According to Abdel-Nour et al. [9], *L. pneumophila* in biofilms is extremely resistant to biocides. Chemical stressors can cause the organism to enter a viable but non-culturable (VBNC) state. This makes accurate laboratory assessment of the bacterium complex as it must be co-cultured with amoeba to reverse the VBNC state. With false negatives in *L. pneumophila* laboratory testing of 66% and 28%, respectively, uncertainties of this magnitude highlight the need to use caution in relying on these tests for routine microbiological control of cooling water systems. They should be used for independently validating effective pathogen control in a cooling tower system while day-to-day operation of the system should include proper microbiological control practices and record keeping.

The complexity and interdependent relationships of the multiple organisms in the cooling water microbiome discussed in this report favors immediate measurement and feedback on their collective state. ATP testing provides immediate assessment of biological conditions in the cooling water system without the low sensitivity or over-reporting seen in either dips slides or plating methods [1].

ATP field methods for microbiological testing are immediate, convenient, and more indicative of immediate or evolving conditions. Culture media results are reported as order-of-magnitude values, which are subjective, while ATP instrumental measurement RLU values are approximately linear, lending themselves to a more objective quantification and evaluation. While ATP field testing also has practical reliability limitations comparable to culture methods, the exact RLU values are unimportant, except to indicate how the system is impacted if it drifts outside an effective range of microbial control with natural inhibitive chemistry.

**Figure 4:**
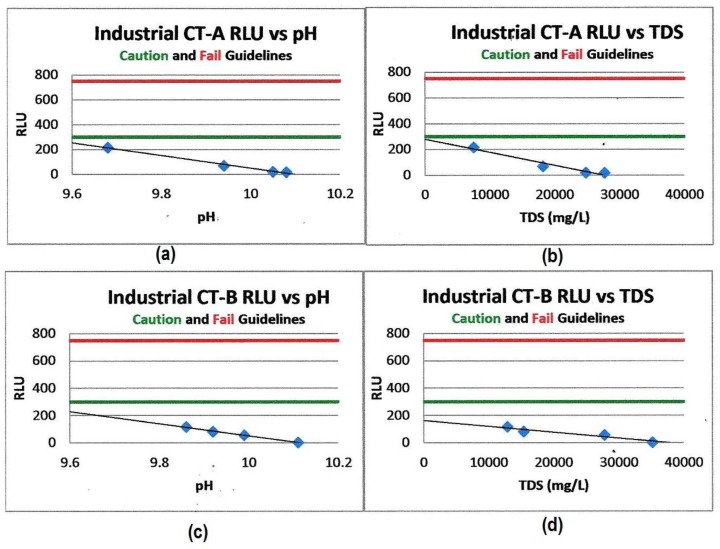
Cooling tower water planktonic bacterial RLU (ATP) values vs. pH and TDS. (**a**) Shows RLU vs. pH for CT-A. (**b**) Shows the RLU vs. TDS for CT-A. (**c**) Shows RLU vs. pH for CT-B. (**d**) Shows RLU vs. TDS for CT-B. The linear inhibitory effects of increased pH and TDS vs. ATP (RLU) values measured in two industrial cooling tower systems [1], the green acceptable control limit line at 300 RLU and the red out-of-control limit line at 800 RLU, demonstrate reduced RLU (ATP) values and therefore the reduced microbiological masses in both systems as the pH and TDS values increase [1].

Rahimian-Pour and Anderson [1] report the reduction in RLU values for two industrial cooling tower systems using this high-cycle water treatment technology. Elevated pH and TDS were shown to synergistically enhance each other’s effectiveness in reducing the amplification of bacterial growth (Figure 4).

Concurrent, on-site plate count testing and ATP testing verified that controlling the pH and TDS chemistries generates a synergistic effect producing cooling water systems with planktonic CFU/mL counts that were at or below the detection levels of the standard agar dip slide tests [1].

## 5. *Legionella* Outbreak Prevention—Control of Biofilm and Trojan Protozoan Hosts

Sessile forms of many microorganisms can lead to biofilm formation that provides a haven for free-living protozoa [2]. According to Abdel-Nour et al. [9], Protozoa are one of the most important groups of microorganisms supporting the widespread propagation and survival of *Legionella* bacteria, and biofilms provide a protective environment for the proliferation of protozoa. Jjemba states [10] that protozoa and biofilms are symbionts that have been found to be integral to *Legionella* amplification in cooling water systems and control of this cooperative microbiological environment is key to preventing *Legionella* outbreaks. *Legionella* are ingested by the protozoa, primarily amoebae, where the pathogens multiply. The expanding bacterial population is ultimately released into the water environment.

According to Davy and O’Toole [21], one of the most important positioning mechanisms for bacterial biofilm formation is aggregation or attachment. Aggregation enhances cell–cell interaction as well as the sedimentation rate of cells. According to Jjemba [10], cooling system predisposition to *Legionella* growth has also been found linked to low-flow operation or piping dead-legs where sediments can collect and biofilm formation is observed. Oliveria et al. [22] state that the opportunistic pathogen, *Pseudomonas aeruginosa*, a common fresh water organism found in cooling towers, generates neutral or anionic polysaccharides that form a biofilm around the developing colony. According to Jjemba [10], the population density of *Legionella* spp. in cooling towers directly correlated with temperature, water pH, and amoeba density. Additional studies have shown a decrease of biofilm-associated *Legionella* when amoeba were absent from the reactor vessel [18]. It is argued that the biofilm gives the bacteria a competitive edge over other microorganisms seeking to establish their colonies in the same ecological niche. Once established, these biofilms also provide a safe haven for a variety of protozoans and certain pathogens that infect humans, including *Legionella pneumophila*. According to Abdel-Nour et al. [9], *L. pneumophila* in biofilms is extremely resistant to biocides. Chemical stressors can cause the organism to enter a viable but non-culturable (VBNC) state.

The authors of this article propose that natural water, cycled to high pH and high TDS levels, effectively prevents normal growth and replication of microorganisms that generate biofilms, thus preventing their biofilms from forming in the first place. According to Rahimian and Anderson [1,6], this inhospitable water environment will prohibit microorganism proliferation, as supported by extremely low ATP test results from cooling tower samples using this control program [1]. The discussions in this report focus on preventative application of this natural antimicrobial chemistry.

According to Barker and Brown [23], *Amoeba* spp. and several other protozoan hosts are the “Trojan Horses” of the microbial world, harboring and protecting several human pathogens, including *Legionella pneumophila*, that aid their survival in the environment. Specifically, *L. pneumophila* has gained notoriety because potentially deadly Legionellosis pneumonia results in approximately 5% of those who are exposed to contaminated water aerosols that contain entrained planktonic bacteria. According to Jespersen and Sofaard [24], in a study in Denmark, the low percentage of those exposed who develop pneumonia is unfortunately offset by the 16% to 55% fatality of those who are admitted with the disease, depending upon whether the exposure was from the community or in a hospital. The CDC reports statistics on a different population in the U.S. They state that there is a 10% mortality rate among those who are diagnosed with Legionellosis, without indication on how the disease was contracted [15].

Most pathogenic microorganisms that survive in humans are neutrophils [3,4], because of the tightly controlled near-neutral pH required in most bodily fluids required for humans to survive. According to Lengeler, Drews, and Schlegel [4], each organism has a unique niche, and it biochemically struggles or dies when exposed to a pH range significantly outside this niche. An exception, *Helicobacter pylori*, is likely the most common non-neutrophile human bacterial infection and according to Brown [25] it inhabits the stomachs of half of the people on Earth, without symptoms. *H. pylori* can survive in a wide range of pH values and are ubiquitous among humans, therefore such exceptions are moot and not relevant to this review. Logically, if neutrophile environmental water pH is shifted significantly from the neutral range, typical pathogens affecting humans and their host protozoa will not survive. If protozoan hosts react to the inhospitable conditions by reverting to their cyst form, the continuous hostile conditions will prevent their proliferation and effectively isolate the pathogen from humans at sufficient disease inducing concentrations.

Natural antibacterial chemistry, according to Rahimian and Anderson, is lethal to the host protozoa [1,6]. When the host organisms rupture and the protection is removed, the *Legionella* trophozoites find themselves in an inhospitable environment that quickly leads to their demise. The effectiveness of this approach is supported by the data in that there have been no documented *Legionella* outbreaks associated with any cooling tower operated under this high-pH, high-TDS method since its inception over the past 10 years. This likely relates to a simple antibacterial residual control method, basically eliminating tower water wastage.

Barker and Brown [23] list several additional human pathogens that take advantage of protozoan protection. *Listeria monocytogenes*, *Vibrio cholerae*, *Mycobacterium leprae*, and *Salmonella typhimurium*, have all been found alive inside host organisms. Because the host microorganisms and pathogens each contain ATP, monitoring and verifying minimal ATP in cooling water systems assures the absence of a significant concentration of human pathogens and the hosts they depend upon for survival and propagation.

**Figure 5:**
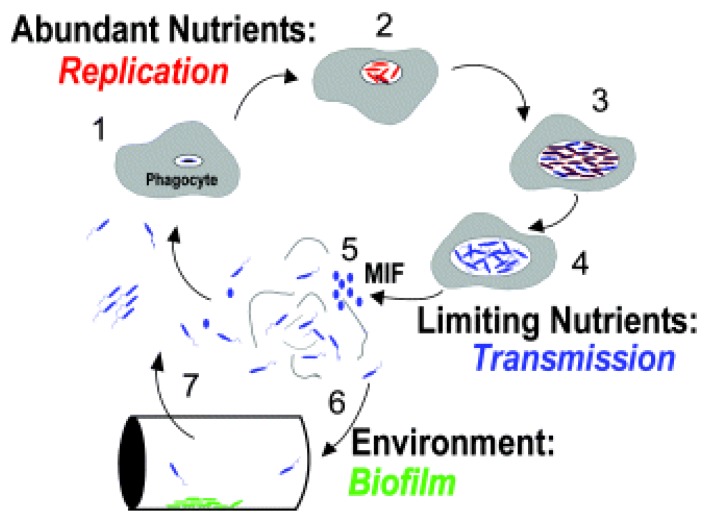
Molofsky and Swanson [26]; the life cycle of *L. pneumophila*. Studies of broth and phagocyte laboratory cultures support the following model for the persistence of *L. pneumophila* in aquatic reservoirs. The notes below match the numbered life stages in the modified Figure 5 above. These descriptions were also modified from the original [26] for clarity. 1. Free-swimming, planktonic *L. pneumophila* that are consumed by phagocytic cells (amoebae or alveolar macrophages) generate vacuoles that protect against lysosomal digestion. 2. When nutrients are present and the internal environment is favorable, intracellular bacteria activate pathways that promote replication. 3. As the environment conditions in the vacuole deteriorate, the offspring stop dividing and develop traits that improve survival in the environment and ingestion by a new phagocytic host. 4. After an extended period, the progeny may develop into a more mature intracellular form (MIF). This cell type is resistant and infectious. 5. The host cell is lysed, and the progeny microbes are released into the water environment. 6. *L. pneumophila* that are not immediately engulfed by a new phagocyte likely initiate biofilms in anthropogenic or natural water systems, where they are resistant to biocides. 7. When free-swimming microbes are engulfed by a new host, the cycle begins again.

The following diagram (Figure 5), from Molofsky and Swanson [26] shows the life cycle of *L. pneumophila*, and the death cycle of infected amoeba. The numbered descriptions for each step in the cycle were modified from the original for clarity. Normally, amoebae phagocytize food particles, including bacteria, and subsequently digest them in low-pH food vacuoles. Chemical signals from the *L. pneumophila* interrupt the normal acidic enzyme digestive stages and allow *L. pneumophila* to continue their amplification in a protected environment. If the environment surrounding the amoebae becomes hostile, the predatory protozoan may revert to a protective cyst. Normally, the encysted form revives when the environment becomes more hospitable. Whether the amoebae remain active or have become a more resistant cyst, if the engulfing predators have had their assets consumed by the now populous parasite *Legionella*, their enclosing membrane ruptures, releasing perhaps thousands of the *L. pneumophila* into the environment.

## 6. Biofilm Inhibition by Polyvalent Metal Ion Concentration Reduction

According to Abdel-Nour et al. [9], iron is required in low concentration for *L. pneumophila* growth and proliferation. However processes required to eliminate such trace quantities of iron from the water environment to control bacteria and pathogens is impractical, as iron is ubiquitous to the water, air, and anthropogenic systems.

Abdel-Nour et al. [9] state that both surface adherent and floating biofilms in anthropogenic water systems provide safe havens for the *Legionella* life cycle. Physio-chemical parameters, such as the common high residuals of divalent cations (Ca^2+^ and Mg^2+^) in most waters, also enhance the attachment of biofilms to surfaces. He also notes that high-carbon sources provide nutrients for the growth of biofilms in aqueous environments.

Comparable studies in both industrial water systems and marine environments found biofilm growth response to be dependent upon the presence or absence of sufficient hardness minerals in the water [9,10]. The research has shown that calcium ions must be present in sufficient concentration for biofilm to form [8]. Although not necessary for microorganism control, this is an additional benefit to using soft makeup water in cooling tower systems and may be used as a mechanism to prevent biofilm accumulation.

Guvensen and Ozdeimer [11] report, that when grown in approximately 7500 mg/L soluble salt growth medium, biofilm formation on coupons increased proportionally to the addition of 0, 100, 250, and 500 μM (0, 4, 10, and 20 mg/L) Mg^2+^. Likewise, similar additions of Ca^2+^ caused a marked increase in *S. paucimobilis* biofilm formation. Comparably, it was also observed that the same concentrations of Ca^2+^ and Mg^2+^ had no impact on free-living (planktonic) *S. paucimobilis* cells in the medium. Hence, the formation of biofilm adherent (sessile) cells was significantly enhanced with increases in Mg^2+^ and Ca^2+^ concentrations.

**Figure 6:**
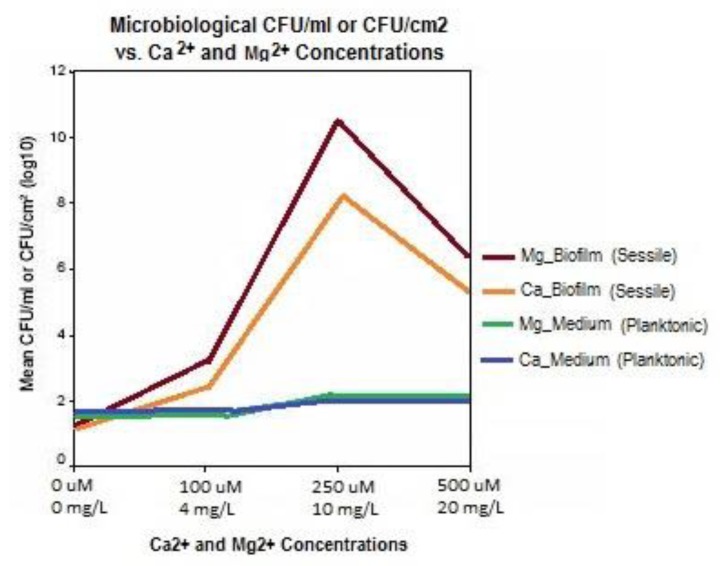
As calcium (Ca^2+^) is added to Tryptone Soya Broth (TSB) inoculated with biofilm forming bacterial pathogen, *S. paucimobilis*, the CFU/ml count goes up significantly for sessile organisms on the growth plates but the free-living bacteria are not significantly affected, indicating a positive correlation between calcium concentration in the water and biofilm formation. (Graph generated with data from [11]).

Guvensen and Ozdeimer [11] also observed that calcium and magnesium cations may directly initiate biofilm formation through electrostatic interactions between the divalent cations and the biofilm, and indirectly by modifying the physiological attachment processes of bacteria. Magnesium (Mg^2+^) has shown to be the molecular activator of key enzymatic biochemical reactions in living cells. Mg^2+^ is credited as one of the intracellular elements and participates in enzyme catalysis, which results in such diverse roles as charge neutralization, structure stabilization, and control of osmotic pressure (Figure 6).

According to Patrauchan [12], under uniform conditions approximating sea water (26,000 mg/L TDS), in Minimum Marine Medium (MMM), variable calcium concentration appears to directly influence thicker bacterial biofilms, primarily through ionic cross-linkage of the extracellular matrix material. This matrix material is typically composed of negatively charged polysaccharides, and the polysaccharide cross-linking with calcium and magnesium ions forms a binding extracellular gel for biofilms. Calcium linkage in these proteins may also play a prominent role in bacterial adhesion to a surface. Bacterial regulatory processes are probably also strongly influenced by calcium ions. In summary, Ca^2+^ does not affect the growth rate of *Pseudoalteromonas* spp. in planktonic culture, but affects biofilm-associated growth on both hydrophobic and hydrophilic surfaces.

**Figure 7:**
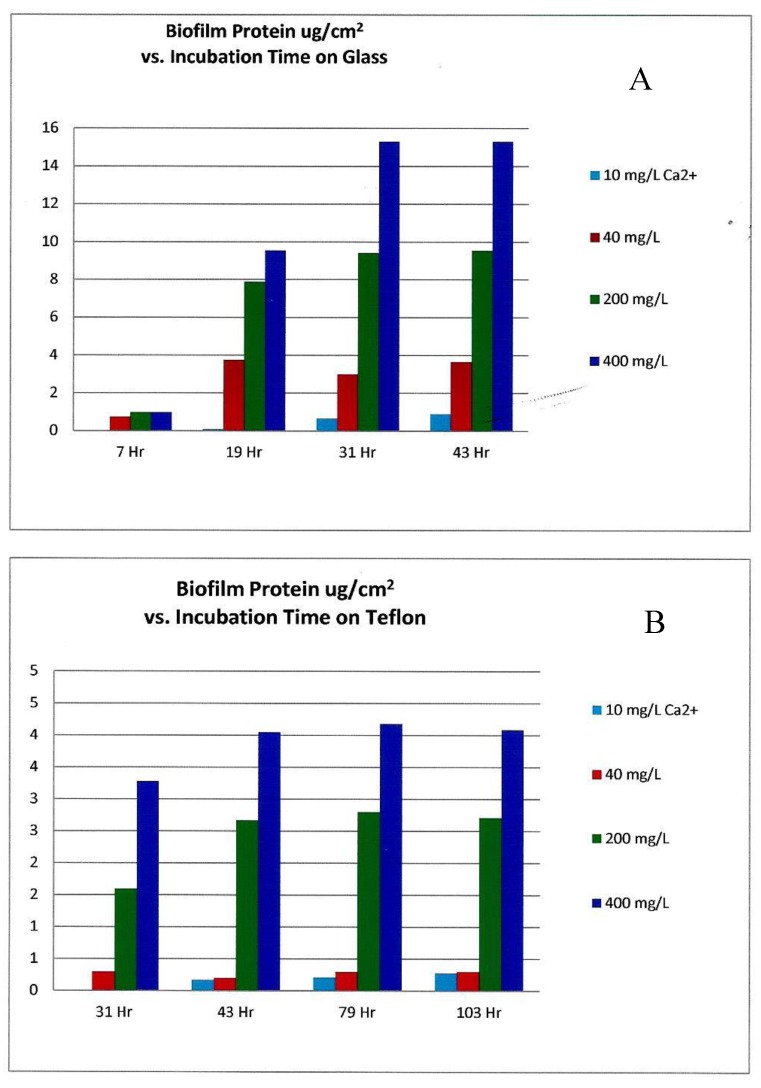
As 10, 40, 200, and 400 mg/L of calcium (Ca^2+^) is added to minimum marine medium (MMM) inoculated with biofilm forming bacteria, the total cellular protein of *Pseudoalteromonas* spp. biofilms associated with (**A**) glass and (**B**) Teflon surfaces increased with time, directly with the increasing concentration of calcium ion. (Graph generated with data from [12]).

The experimental growth solutions had somewhat higher calcium concentrations in the sea water simulation, ranging from 0 mg/L to 400 mg/L as Ca^2+^ ion [12], as opposed to the fresh water experiment which had 0 to 20 mg/L as divalent ion [11]. While these two independent experimental investigations did not appear to compare or contrast results, the peak biofilm growth in the fresh water experiment appeared to occur at 10 mg/L Ca^2+^ concentration, while Patrauchan’s sea water model [12] required a 40× or greater Ca^2+^ concentration for peak growth (Figure 7). This may be due to the significantly higher concentration of monovalent metal ions (Na^+^) in the MMM growth medium experiment [12]. As found with ionic salts competition, the higher molar concentration of sodium ions may have competed for the cation cross-link sites in the polysaccharide matrix as well the enzyme activation sites within the microorganisms. This competition would further inhibit bacterial access to Ca^2+^ and Mg^2+^ ions in high TDS cooling tower water, making the chemistry even more inhibitive to biofilm formation.

From a review of the chemical components in the MMM [12] and the Tryptone Soya Broth (TSB) [11] used in these independent studies, there were no precipitating ionic pairs present. One could conclude that the biofilm forming bacteria were able to extract and metabolize Ca^2+^, and then adhere to the respective experimental growth surfaces without the presence of precipitated calcium, magnesium, or iron at the surface. The microbes were able to perform their metabolic functions using dissolved, waterborne Ca^2+^ ions. Thus we can conclude scale or corrosion deposits are not required for biofilm formation, as these organisms are able to create their polysaccharide matrix in either case. Pretreatment removal of these metal ions from water creates an environment that undermines and inhibits biofilm formation, defeating a formidable survival mechanism used by the microbiological population.

Comparable indication of the role of calcium, magnesium, and polyvalent metals in the formation of biofilms was also indicated in studies hosted by CDC for medical applications. Donlan [27] states that gram-negative bacteria, such as *L. pneumophila*, generate neutral or anionic polysaccharides. This property is important because it predisposes the association of the polysaccharides with divalent cations such as calcium and magnesium. The resulting cross-linkage of the polymer strands provides greater binding force in an established biofilm.

One may conclude from the results in these three independent studies that the concentration of calcium and magnesium was a significant contributory, dependent variable that overshadowed other inhibitive effects such as TDS concentration or the specific biofilm forming organism in their respective environments. Eliminating calcium and magnesium ions from cooling tower water appears to deprive some categories of bacteria the ability to adhere to surfaces and therefore prevent or greatly inhibit bacterial slime formation, as indicated in the prior studies. This process also removes the biofilm safe haven for *Legionella*.

**Figure 8:**
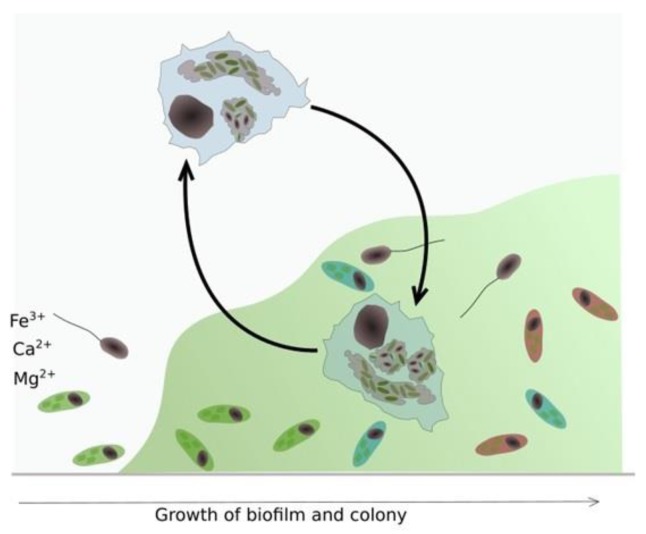
As the biofilm becomes more established, the microbial diversity increases which is a good environment for *Acanthamoeba* spp. *Acanathamoeba* can survive in the trophozoite and amoeboid cyst forms inside and outside biofilm. (Figure 8 generated by Daniel Brouse).

Figure 8 below, shows how biofilms can develop under conditions conducive to their growth. Cooling towers are designed to conduct heat from a process exchanger and discharge it to the atmosphere through evaporation, but also serve as ideal incubators for microorganism and biofilm propagation.

**Figure 9:**
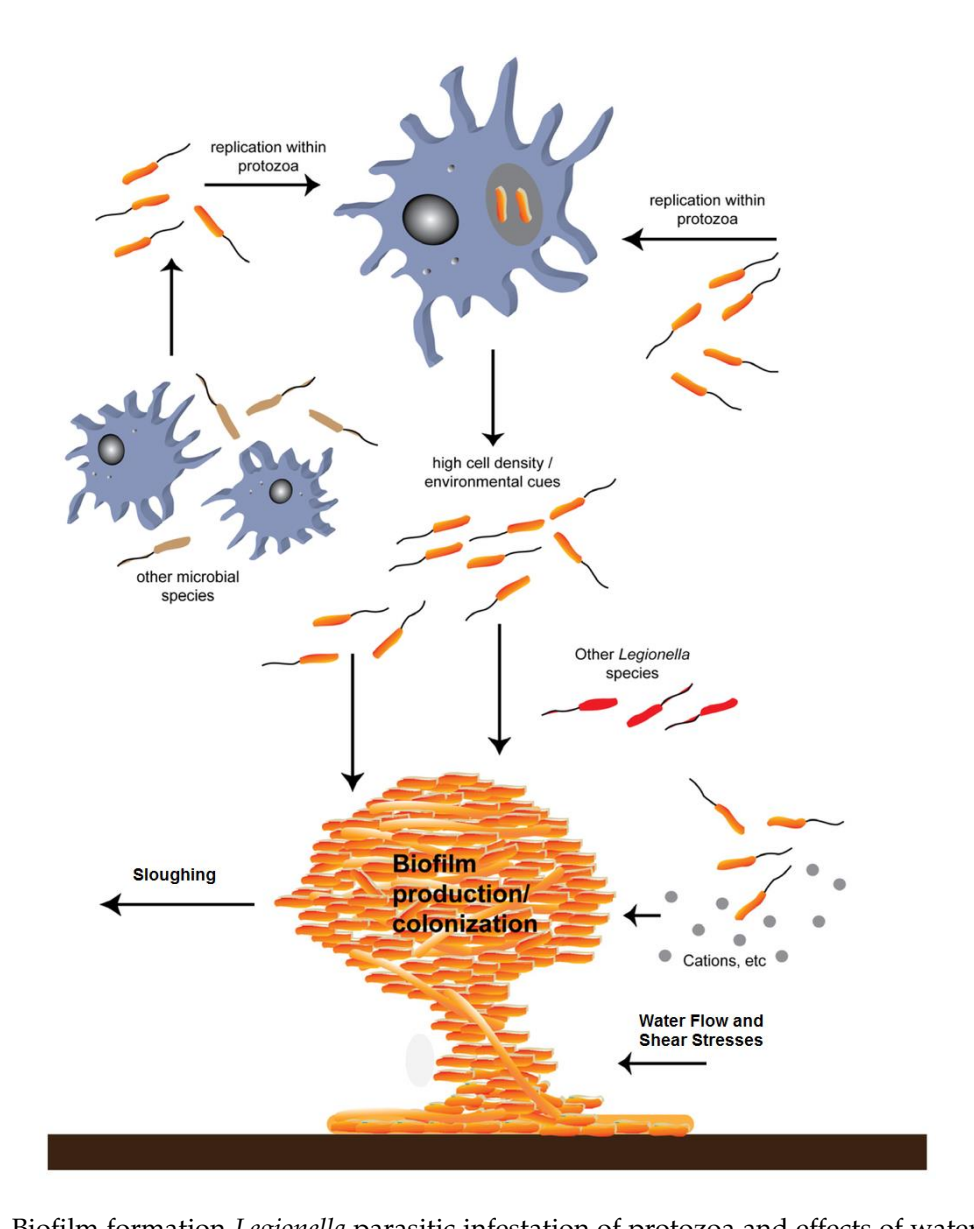
Biofilm formation-*Legionella* parasitic infestation of protozoa and effects of water flow on sloughing and redistribution of planktonic *L. pneumophila* in cooling water systems. (Image modified from [9]).

Figure 9 below, adapted from Abdel-Nour [9], depicts biofilm formation under the influence of cooling water circulating rates that generate typical flow velocities up to 10 feet-per-second through the system. The circulation normally keeps the process operating efficiently. When cooling tower systems are taken offline or otherwise lose their circulation, low flow or no flow conditions result [1]. This is one of the conditions under which bacteria proliferate. In the presence of calcium ions, insoluble polysaccharides form a biofilm matrix in which a growing variety of additional microorganisms take up residence. This becomes a good hunting area for phagocytic amoeba species. *Legionella* are engulfed and thus become amoebic parasites in a protected environment that, without intervention, may persist for decades. High pH and high TDS do intervene and interfere with the metabolism of the hosts, precluding their surviving the inhospitable chemistry environment. Without protective hosts, the parasites become vulnerable to the same antibiotic effects of the circulating water [1].

**Figure 10:**
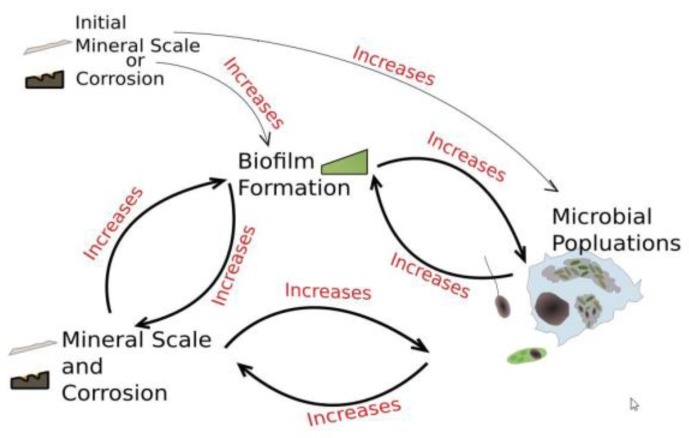
A horrible synergy—Control of scale and corrosion is part of microbial control. (Figure 10 generated by Daniel Brouse).

Additionally, as depicted in the following diagram (Figure 10), scale deposits or corrosion product formation generate safe harbors for under-deposit microbiological growth and replication. This process becomes a self-perpetuating loop with additional microbe populations available to colonize newly-formed deposits of either type.

## 7. Biofilm Control Summary

The biofilms formed by various bacteria can be prevented by removing divalent ions (Ca^++^ and Mg^++^). Concurrently, modifying the water chemistry environment to a pH range between 9.5 and 10.0 and TDS above 20,000 mg/L, will minimize all bacterial propagation and survival. If one prevents biofilms from forming, the habitat for protozoa is removed and the intracellular protection and breeding ground for *L. pneumophila* is eliminated. Pathogen control is the default condition of such chemistry. Ancillary benefits may also be derived with use of soft water in domestic hot water systems to avert biofilm formation, thereby reducing potential for propagation and survival of pathogens such as *Legionella*.

## 8. Conclusions

The antibacterial effects of natural water chemistry concentrated in cooling towers have been demonstrated to be effective while also eliminating use and discharge of toxic agents. The method also provides ancillary benefits for scale and corrosion reduction that mitigate biofilm and microbiological growth, in addition to reducing associated energy penalties, water consumption, and discharge. Natural antibacterial water chemistry can thus be employed to reduce global dependence on toxic antibacterial agents and their harmful impact when discharged into the environment. Such ecologically sustainable practices can potentially reduce the environmental impact to the natural microbiome, plants, animals, and humans, and reduce urgent concerns with the development of pathogen resistance to antibacterial agents and antibiotics currently in use.
